# Selective deficit of second language: a case study of a brain-damaged Arabic-Hebrew bilingual patient

**DOI:** 10.1186/1744-9081-5-17

**Published:** 2009-03-12

**Authors:** Raphiq Ibrahim

**Affiliations:** 1 Department of Learning Disabilities, University of Haifa, Haifa, Israel; 2 Cognitive Neurology Unit, Rambam Medical Center, Haifa, Israel

## Abstract

**Background:**

An understanding of how two languages are represented in the human brain is best obtained from studies of bilingual patients who have sustained brain damage. The primary goal of the present study was to determine whether one or both languages of an Arabic-Hebrew bilingual individual are disrupted following brain damage. I present a case study of a bilingual patient, proficient in Arabic and Hebrew, who had sustained brain damage as a result of an intracranial hemorrhage related to herpes encephalitis.

**Methods:**

The patient's performance on several linguistic tasks carried out in the first language (Arabic) and in the second language (Hebrew) was assessed, and his performance in the two languages was compared.

**Results:**

The patient displayed somewhat different symptomatologies in the two languages. The results revealed dissociation between the two languages in terms of both the types and the magnitude of errors, pointing to aphasic symptoms in both languages, with Hebrew being the more impaired. Further analysis disclosed that this dissociation was apparently caused not by damage to his semantic system, but rather by damage at the lexical level.

**Conclusion:**

The results suggest that the principles governing the organization of lexical representations in the brain are not similar for the two languages.

## Background

A better understanding of how two languages are represented in the human brain is best obtained from studies of bilingual patients who have sustained brain damage. The primary goal of the present study was to determine whether one or both languages of an Arabic-Hebrew bilingual individual are disrupted following brain damage [1,2]. Analysis of language impairment has up to now been restricted largely to English and other western European languages. This descriptive study provides an opportunity to observe how this deficit is manifested in two languages whose linguistic structures do not differ substantially. In addition, it enables us to gain a better understanding of the nature of selective impairment of phonological, orthographic, lexical, and grammatical structures in bilingual individuals. Since the linguistic structures of Indo-European languages differ substantially from those of Arabic and Hebrew, this study also presents a major challenge to psycholinguists seeking to understand the dynamics of language processing and language acquisition. The large amount of data obtained in this study is likely to provide clearer and more comprehensive answers to the research question.

### The neural basis of bilingualism

The question of how multiple languages are represented in the brain remains unresolved. On one hand, some data point to a single neural representation for multiple languages [3,4] This is called the "linguistic domain" approach. On the other hand, some data indicate that bilingual individuals could have distinct cortical language areas [5,6]. This has been termed the "language-membership principle". According to this approach, first language (L1) and second language (L2) representations would, at least to some extent, be sustained by different brain areas, because they show different language membership values.

Cases of selective aphasia and other findings obtained by neuroimaging techniques demonstrate a dissociation between multiple language representations in the cognitive system of the brain [6]. In their functional magnetic resonance imaging (fMRI) study of French-English bilingual individuals, Dehaene et al. found dissociation between cortical areas involved in French (L1) and in English (L2) [6]. During presentation of L1, for example, the left superior temporal sulcus and the superior and middle temporal gyri showed consistent activation across subjects. Thus, according to the language-membership principle, a bilingual aphasic individual is likely to show selective recovery in one language while the other is lost (see [7,8]). The classical model assigns language functions to two regions in the left hemisphere of the brain, namely the inferior frontal region and the temporoparietal region. Injuries within the general boundaries of these cortical areas have resulted in clinically and linguistically different aphasic syndromes, referred to as Broca's aphasia (agrammatic) and Wernicke's aphasia (paragrammatic).

In recent years, the above localization theory of language has lost much of its categorical power. Some researchers have provided evidence that certain components of language (for example semantics) are localized in the right hemisphere [9,10]. Additional evidence comes from epileptic bilingual patients who performed a picture-naming task in L1 and L2 while different brain areas were being stimulated (e.g, [11]). Lucas et al. suggested that both overlapping and distinct brain regions are involved in the representations of the different languages of a bilingual individual [11]. Furthermore, studies of brain activity have shown that the same brain regions are responsible for the representation of both languages of a bilingual individual, regardless of the degree of similarity between the languages [12]. According to this view, organization of the lexical representations of L1 and L2 in a bilingual individual would be governed by variables such as grammatical class and semantic category, regardless of language membership. Klein et al. compared the cerebral organization of two typologically distant languages, English and Mandarin Chinese [12]. The subjects, proficient in both languages, had acquired their L2 during adolescence. Mandarin was chosen because it differs from English in its specified use of pitch and tone. The study examined the influence of linguistic structure on cerebral blood flow patterns in subjects performing a noun-verb generation task. The task conditions consisted of repeating nouns in Mandarin, repeating nouns in English, generating a verb for a noun in Mandarin, and generating a verb for a noun in English. Overall, the pattern of cerebral blood flow increase seen in response to L1 was strikingly similar to that seen for L2. This finding led to the conclusion that in fluent bilingual individuals who use both languages in daily life, lexical search utilizes common cortical areas. More recently, results based on event-related (ER)-fMRI showed a shared neural mechanism for the processing of native and second languages [13]. Moreover, Illes et al. examined brain activation in bilingual participants who had sequentially learned English and Spanish (or Spanish and English) [14]. The participants had become fluent in L2 a decade after acquiring L1, and at the time of the study they were proficient in both languages. Subjects were presented with 480 concrete and abstract English nouns and their Spanish translations, and were required to make semantic and non-semantic decisions about those words. The results showed that semantic activation for both languages occurs in the same cortical locations. Furthermore, no differences in activation were observed when semantic judgments in English and Spanish were directly compared. On the basis of the resolution provided by fMRI, the researchers suggested that semantic processes for the two languages in the bilingual brain are mediated by a common neural system. They concluded that learning a new language after puberty does not require the addition of a new semantic processing system or the recruitment of new cortical regions.

A third hypothesis postulates that both linguistic domain and language membership affect the way in which L2 information is represented in the brain.

With regard to the issue of cognitive representation of the two languages in bilingual individuals, several psycholinguistic models have been proposed. Current models of lexical access in bilingual speakers typically assume that the semantic system in such individuals is shared by the two languages [15-18]. In other words, each semantic/conceptual representation is connected to its corresponding set of lexical nodes common to the two languages. Although some researchers (e.g.,[19]) claim that conceptual representations are language dependent, recent proposals favor the idea that, at least for common words, bilingual subjects have a unique conceptual store shared by the two languages. If the bilingual individual's semantic system is indeed shared by both languages, this raises a question: is there a spread of activation between the semantic system and the lexical system regardless of the language programmed for response? Researchers are also interested in determining whether activation of the semantic system spreads to both languages. Some authors claim that the two languages are activated in parallel, regardless of the language chosen for production [15,16,20].

Accordingly, some current models follow the principle of general spread of activation and assume that there is parallel activation of the two lexicons. Levelt assumed that concepts are represented as indivisible nodes, and that the nodes corresponding to a concept are linked to the nodes of semantically related concepts [21]. For example, activation of the conceptual node corresponding to a picture (e.g., *bird*) "spreads" some activation to semantic representations that are associated with it (such as *tree*, *aeroplane*). Other models [22,23] are based on the assumption that concepts (e.g. *canary*) are represented as a bundle of semantic features (*bird, can fly, two legs*) and that activation of a given concept (e.g., *bird*) activates part of the semantic representation of related concepts (e.g., *penguin*) because some of their semantic features are shared. Regardless of their specific mechanisms, these two proposals share the assumption that in the course of naming a picture, several semantic representations are activated to some degree. This is either because semantic representations are interconnected or because they share several semantic features.

Other psycholinguistic models are based on the assumption that words of each language are represented separately at the lexical level, and are connected indirectly via a common semantic system that is accessed independently from each lexicon [24,25]. In neurolinguistic terms, these models suggest that separate but overlapping regions are involved in the processing of more than one language.

Two factors complicate the debate on cerebral organization in bilingual people: first, the time of acquisition of the native and the second languages (early or late bilingual), and second, the degree of fluency in these languages (high or low proficiency). Kim et al. compared levels of proficiency in both languages of subjects who had been exposed to both formal education and colloquial exposure [26]. They suggested that the level of proficiency is a critical determinant of brain activation patterns in language tasks. In other words, they suggested a common cortical representation for L1 and L2 when both languages are acquired early, implying high levels of proficiency in both languages.

The patient (MH) described below, an Arabic-Hebrew bilingual man who had acquired Hebrew by exposure to both formal education and colloquial setting after the age of 9, evinced a dissociation between his ability both to perceive and to produce his second language (Hebrew) after sustaining brain damage. To the best of my knowledge, this report is unique in that this problem of language organization has not been previously studied in relation to these two particular languages. The report is preceded by a summary of the similarities and differences between Arabic and Hebrew.

### Arabic and Hebrew – background and characteristics

Arabic and Hebrew are both Semitic languages, and their words have similar morphological structures. Regardless of whether these words are based on inflectional or derivational forms, the morpheme-based lexicon of these families implies the existence of roots and templates (word patterns). Roots are recognized as autonomous morphemes expressing the basic meaning of the word. Roots are abstract entities that are separated by vowels that add morphological information (e.g., in Arabic, the perfective /a-a/ in *daraba *'hit', or the passive /u-i/ in *duriba *'was hit'; in Hebrew, the perfective /a-a/ in *lakah *'took', or the passive /ni-a/ in *nilkah *'was taken'). Other researchers have defined both of these Semitic languages in terms of nonconcatenative, highly productive derivational morphology [27]. According to this definition, most words are derived by embedding a root (generally trilateral) into a morpho-phonological word pattern, where various derivatives are formed by the addition of affixes and vowels. Also, in both Arabic and Hebrew there are four letters which also specify long vowels, in addition to their role in signifying specific consonants (in Arabic there are only three – a, u, y ا و ي). However, in some cases it is difficult for the reader to determine whether these dual-function letters represent a vowel or a consonant. When vowels do appear (in poetry, children's books and liturgical texts), they are signified by diacritical marks above, below, or within the body of the word. Inclusion of these marks specifies the phonological form of the orthographic string, making it completely transparent in terms of orthographic/phonologic relations.

With regard to semantics, the core meaning is conveyed by the root, while the phonological pattern conveys word-class information. For example, in Arabic the word *takreem *consists of the root *krm*, whose semantic space includes things having to do with respect. and the phonological pattern ta--i. The combination results in the word 'honor'. In Hebrew, the word *sifra *consists of the root *sfr*, whose semantic space includes things having to do with counting. and the phonological pattern -i--a, which tends to occur in words denoting singular feminine nouns, resulting in the word 'numeral'. Because the majority of written materials do not include the diacritical marks, a single printed word is often not only ambiguous between different lexical items (this ambiguity is normally solved by semantic and syntactic processes in text comprehension), but also does not specify the phonological form of the letter string. In their unpointed form, therefore, Hebrew and Arabic orthographies both contain only limited amounts of vowel information and include large numbers of homographs. Compared to Hebrew, Arabic has a much larger number of homographs, and consequently is much more complicated.

Despite the similarity between the two languages, there are major differences between them. First, Arabic has a special case of diglossia that does not exist in Hebrew. Literary Arabic is universally used in the Arab world for formal communication and is known as "written Arabic", also called "Modern Standard Arabic". Spoken Arabic, which appears partly or entirely in colloquial dialect, is the language of everyday communication and has no written form. Although they share a limited subgroup of words, the two forms of Arabic are phonologically, morphologically, and syntactically different. This added complexity is found in several characteristics that occur in both orthographies, but to a much larger extent in Arabic than in Hebrew, where the orthography is restricted to letters, diacritics and dots. In both orthographies some letters are represented by different shapes, depending on their placement in the word. Again, this device is used much less in Hebrew than in Arabic. In Hebrew there are five letters that change shape when used in the final position: (כ-ך, פ-ף, צ-ץ, נ-ן, מ-ם). In Arabic, 22 of the 28 letters in the alphabet have four shapes each (for example, the phoneme /h/ is represented as: ـه ٬ه ـهـ ٬ هـ٬). Thus, the grapheme-phoneme relationships in Arabic are quite complex, with similar graphemes representing quite different phonemes, and different graphemes representing the same phoneme. In Hebrew, for example, dots occur only as diacritics to mark vowels and as a stress-marking device (dagesh). In the case of three letters, this stress-marking device (which does not appear in unvowelized scripts) changes the phonemic representation of the letters from fricatives (v, x, f) to stops (b, k, p for the letters פ ק ב respectively). In the unvowelized form of the script these letters can be disambiguated by their place in the word. In Arabic, dots are more extensively used; many letters have similar or even identical structures and are distinguished only on the basis of the existence, location, and number of dots (e.g., the Arabic letters representing /t/ and /n/ ٬ن, ت) become the graphemes representing /th/ and /b/ (ب, ث) by adding or changing the number or location of dots.

Many studies have demonstrated that bilingual individuals do not recognize written words in exactly the same way as monolingual individuals do. It was shown, for example, that visual word identification in L2 is affected by the native language of the reader (e.g.,[28]). However, the opposite is also true: knowledge of L2 can affect the identification of printed L1 words [29]. In comparative studies of different languages, it is customary to compare both speech and writing systems. Thus, in a comparison of Arabic and Hebrew reading, we compare examples of two related language families (Semitic languages) that are similar in their morphological structure but radically different in their orthographic and phonetic systems. Recent morphological studies of the Hebrew [30] and Arabic languages [31] support the assumption that roots can be accessed as independent morphological units. In the area of speech perception, some authors have reported differences in the phonetic perception of L1 and L2 between native (for reviews see [32]) and nonnative speakers [33].

### Case report

M.H. is a 41-year-old, right-handed, male high-school biology teacher. Born in Israel, he is a native speaker of Arabic and acquired Hebrew language in the 4^th ^grade. He has used Hebrew in academic, professional, and private settings, and premorbidly (as reported by his brother) his Hebrew competence was very high. He graduated from a university where the language of instruction is Hebrew, having passed a Hebrew proficiency examination upon university entrance.

**Figure 1:**
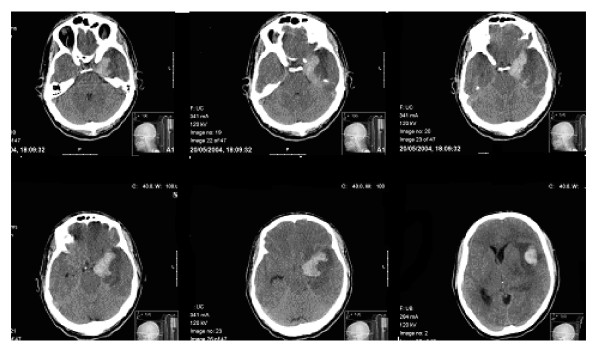
**Preoperative computerized tomography(CT) scan**. The scan shows intracranial hemorrhage over the left temporal lobe and right frontal subdural hemorrhage compressing the central line of the brain contralaterally.

In May, 2004 he was taken to the local hospital with sudden onset of fever and confusion. Upon initial examination he was febrile, confused and disoriented, and was promptly referred to the regional hospital (Rambam Medical Center, Haifa). On the 3^rd ^day in hospital his cerebrospinal fluid, tested by polymerase chain reaction, was positive for *Herpes simplex *virus, and antiviral therapy (Zovirax) was initiated. On the 5^th ^day he suddenly exhibited high-grade headache, vomiting, and disturbance of consciousness. Radiological findings disclosed an acute, massive intracranial hemorrhage in the left temporal lobe, compressing the central line of the brain contralaterally (Figure 1). On the same day he underwent left temporal craniotomy to removal the massive lesion, after which computed tomography scanning demonstrated moderate hemorrhage and encephalomyelitis in the left temporal lobe and right frontal subdural hemorrhage. An association between subarachnoid hemorrhage and infection has been reported by researchers although the notion that viral infection may be an important factor in intracranial aneurysm formation and subsequent rupture is a matter of debate [34]. The patient showed uneventful initial recovery, with low-grade fever for only 12 hours immediately after surgery. A few days after the operation he became lethargic and was sent for rehabilitation to Beit Levinstein Hospital, where he was hospitalized for 2 months. During this period he experienced an acute onset of a neurological deficit, and developed epileptic status with left temporal focus, as well as amnestic aphasia.

**Figure 2:**
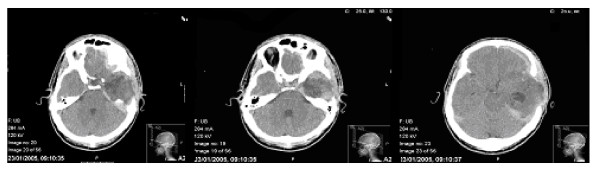
**Postoperative computerized tomography(CT) scan**. A follow-up CT scan 9 months later shows a modified decompressive craniotomy for removal of hematoma.

Upon admission to Beit Levinstein MH was active, cooperative, and well oriented in place, situation, and time. Visual fields and auditory abilities were intact. His spontaneous language production was non-fluent, with grammatical disruptions and frequent anomic states. Two tests were administered: a subtest (Fluency) from the Western Aphasia Battery (WAB) [35] and the Boston Naming Test (BNT) [36] in Arabic and Hebrew. The language status that emerged from these tests was consistent with mild to moderate amnestic aphasia [37]. Follow-up computed tomography (CT) 9 months later showed modified decompressive craniotomy for removal of hematoma (Figure 2). The neuropsychological tests for the present study were conducted in January 2006, i.e., after the rehabilitation period.

## Results

### Repetition, naming and comprehension

A dissociation was observed between the patient's performance in Arabic and in Hebrew. A paired *t*-test was used to estimate their relationship to one another by examinining whether the mean performance in Arabic (L1) is equal to the mean performance in Hebrew (L2). In Arabic, MH exhibited almost fluent speech, whereas in Hebrew his speech was characterized by word-finding pauses and paraphasic errors. A paired-samples *t*-test disclosed a significant difference between his performance in the two languages (*t*_(2) _= 13, *p *< 0.01). Furthermore, he exhibited only limited disturbances in auditory comprehension in Arabic (*t*_(3) _= 5.7, *p *< 0.01), but significantly more disturbances in Hebrew. In written language, he encountered more problems in reading and writing in Hebrew than in Arabic.

To summarize, in Hebrew MH exhibited non-fluent speech with anomia and disturbances in auditory comprehension, but without difficulty in repetition. From the results shown in Table 1 it can be seen that patterns of impairment emerged in both languages, with the more severe impairment in Hebrew. In addition, some abilities were found to be preserved in single-word reading as well as in writing to dictation in Hebrew. Following intensive language therapy in both Arabic and Hebrew for 3 months, he showed significant improvement in both languages and especially in Arabic. The improvement in Arabic was in all linguistic abilities. In Hebrew there was mild improvement in spontaneous speech and auditory comprehension, but naming ability remained unchanged. Currently his speech in Arabic is fluent and grammatically correct, but with occasional paraphasias and pronounced word-finding difficulties. His reading and writing abilities have improved significantly in Arabic only.

**Table 1:** Western Aphasia Battery scores indicating degrees of language impairment in Arabic and in Hebrew

**Subtest**	**Arabic**	**Hebrew**
Fluency	8/10	4/10**
Comprehension	7.8/10	5.5/10**
Repetition	10/10	8/10
Naming	7/10	3/10*

*Equivalent to less than the 5th percentile; **equivalent to less than the 10th percentile.

MH's most pronounced aphasic symptom, both initially and residually, was a marked difficulty in confrontation naming in both languages. At first (at least during August and September, 2004), he exhibited an almost typical pattern of amnestic aphasia [38]: His initial score on the BNT was 15/60 (> 2 SD) in Arabic and 25/60 (> 1 SD) in Hebrew. During treatment there was a change in the clinical picture of his anomic disorders: in Arabic there was an improvement (32/60) (< 1 SD), whereas his anomic impairment in Hebrew slightly deteriorated (22/60) (> 2 SD). Table 1 and Table 2 shows that naming abilities were impaired in all modalities and in all types of naming tasks. However, these deficits were not equivalent in the two languages, and Arabic was the more productive. Phonemic priming was effective and the patient's performance improved if he was presented with more than one syllable. Treatment was followed by a gradual but significant improvement in auditory comprehension (including single-word comprehension).

**Table 2:** Scores obtained on naming tasks in Arabic and Hebrew

Tasks	Arabic	Hebrew
Category generation task	7	3*
Letter generation task (B)	7	2*
Tactile naming	7/10*	3/10*

*Equivalent to less than the 5th percentile; *equivalent to less than the 10th percentile.

In the category generation task, the patient was asked to name as many members of a specified semantic category as possible in 1 minute. The list of categories included animals and fruits. In the letter generation task *(B)*, he was asked to name all the words he could think of that begin with the letter *B*. In the tactile naming task, using the same 10-item set of household objects whose use the patient had been able to communicate by gesture, the patient's ability to name an object by touching it was compared with his visual naming ability.

### Visual abilities

To rule out the possibility that the patient's symptoms were caused by the right frontal hemorrage, his performance was assessed on tasks that demonstrate visuospatial and frontal difficulties. The results showed that his visual ability was good (Table 3). He also demonstrated good copying and construction abilities on the Rey Complex Figure test, achieving a score of 33/36 [39]. His capacity for non-verbal abstraction, examined by the Wisconsin Card Sorting Cards test (WCST), was close to normal for his age, consistent with his intact visual perception and reasoning skills.

**Table 3:** Performance on tests of visual ability

**Task**	**Results**
Matching pictures	10/10
Matching shapes	10/10
Matching letters*	10/10
Matching words*	10/10

* In both languages

### Phonological/phonetic abilities

MH was given three auditory tasks, as described by Luria (1970): (a) counting the letters in individual spoken words, (b) counting syllables in an individual spoken word, and (c) synthesizing words from individually pronounced letters (i.e., recognizing an auditorally spelled word) [40]. In all of these tests the examiner's mouth movements were hidden from the patient's view. The results are recorded in Table 4. The patient's performance on these tasks was found to be dependent on word length, with better performance on short words (three to five letters). Both Arabic and Hebrew are languages with deep orthography, i.e., they do not have one-to-one correspondence between letters and sounds, because most Arabic and Hebrew vowels are not instantiated as letters. This is probably reflected in the relatively similar performance by MH in both languages. A paired-samples *t*-test showed no significant differences between the results obtained in Arabic and in Hebrew *(t*_(2) _= -1.94, *p = *1.92). It was observed that MH had counted phonemes instead of letters. Interestingly, in naming Hebrew phonemes MH used the Arabic "popular terms", meaning that he referred to the sounds rather than the names of these letters. For example, when presented with the letter he said [*ba*] instead of [*bet*]. Also, in many cases he counted syllables instead of sounds or letters. His ability to count the number of syllables was intact.

**Table 4:** Performance on auditory tasks reflecting phonological ability

**Task**	**Arabic**	**Hebrew**
Counting letter	16/20	13/20**
Counting syllables	20/20	20/20
Spelled word recognition	8/10	4/10*

*Equivalent to less than the 5th percentile; **equivalent to less than the 10th percentile.

### Reading and writing

When reading aloud in Arabic, MH demonstrated two strategies. In some cases of single and short words he seemed to use a direct visual strategy, immediately recognizing the word. In other cases this strategy was not successful and he turned to letter-by-letter reading, resulting in literal paralexias (for example, the word *a'melat*, "workers," was read as *a'lamat*, which is not a meaningful word) but often he recognized this immediately and corrected it himself. His strategy for reading in Hebrew was similar, but his performance was poor. This is probably attributable to the general inappropriateness of letter-by-letter reading for unvoweled Hebrew (see [41]). His spontaneous writing (in Arabic) was good at the level of single words and word combinations without literal paragraphias. In Hebrew, MH was able to write to dictation only at the level of words with literal paragraphias (for example, the word *mapa*, "map," was written as *maba*, which is not a meaningful word).

## Discussion

After sustaining brain damage, MH displayed somewhat different symptomatologies in his two languages. The results of the standard examination showed that the language impairments in Arabic and in Hebrew differed, and that the disorder was significantly more prominent in Hebrew.

He also progressed differently in the two languages following language therapy, where more progress was made in Arabic. This clinical picture is interesting because Arabic is structurally not very different from Hebrew especially in terms of morphology and syntax. It should also be borne in mind that although Arabic is the patient's native language, his level of competence in the two languages before he sustained brain damage was almost equivalent. In the course of his language treatment, various tests were administered to further clarify the nature of his impairments in the two languages. The initial diagnosis was amnestic aphasia.

An important factor in the case was the localization of the brain injury. MH apparently sustained at least three distinct neurological insults during his hospitalization: herpes encephalitis with intraparenchymal hemorrhage into the left temporal lobe, right frontal subdural hematoma, and epileptic seizures. In addition, he underwent urgent surgical craniotomy of the presumably dominant hemisphere. Because each insult affects separate language networks, it is important in such cases to provide a detailed account of the patient's clinical language deterioration as it relates to the specific insult. Herpes encephalitis is known to involve the bilateral temporal lobes. Among other functions, the temporal lobes are critical for short-term memory consolidation (hippocampus) and naming functions (dominant hemisphere. middle and superior temporal gyrus) [42].

With regard to the other neurological insults, a subarachnoid hemorrhage and possible aneurysm were reported. This potentially aneurismal subarachnoid hemorrhage might be associated with delayed ischemic deficits, vasospasm, and distal thromboembolic events, and hence be related to the language symptoms. For this reason, it is critical to take careful note of the timing and nature of the patient's language deterioration; in this particular case, such monitoring supported the involvement of bilateral temporal lobes. This conclusion is in line with previous published studies of the language organization of the temporal lobe, particularly with respect to the anatomy of the superior and middle temporal gyrus [43].

The modularity hypothesis developed in cognitive science offers a possible explanation of the major aphasia syndromes. That theory posits, for example, that Broca's aphasia is the result of computational deficits which occur within linguistic components that are unrelated to a specific language. Research over the past 30 years has yielded substantial evidence supporting dissociation between languages [6,8,44] and specifically that the performance of aphasic patients on some linguistic tasks may vary across languages. Such findings have contributed to a better understanding of the nature of language representation. In connection with bilingualism, two main hypotheses have emerged. One suggests that representations of L1 and L2 would, at least to some extent, be sustained by different brain areas. The other posits that L2 representations would be organized on the basis of exactly the same principles as those governing L1 organization, and hence that L1 and L2 representations are sustained by the same brain areas. Regardless of language membership, moreover, the lexical representations of the two languages would be governed by variables such as grammatical class and semantic category; thus, this approach does not preclude the possibility that a bilingual aphasic patient might selectively recover one language while the other is lost (see [7,8]).

Our patient's results in the naming tasks suggest that his naming difficulties probably arose as a result of damage to a lexical retrieval mechanism. However, not all linguistic components (such as naming) are necessarily similar in the two languages. As outlined in the Introduction, once the target lexical node is selected the next step in speech production is selection of the word's phonological segments. The dynamics of activation and selection of the phonological component of words varies widely between models. One major difference relates to the extent to which the models implement the principle of spreading of activation between the lexical level and the phonological level. Although the spreading activation principle has been widely adopted when characterizing the dynamics of processing between the semantic level and the lexical level, it is not as widely employed when characterizing processing at the segmental phonological level. According to discrete stage models of lexical access [21,45], activation of phonological properties is restricted to those of the selected lexical node. Furthermore, activation of the phonological properties of words begins only after the target lexical node has been selected. In contrast, cascade models of lexical access [22,46] are based on the assumption that all the lexical nodes activated from the semantic level send proportional activation to their phonological segments. Furthermore, activation of the phonological properties of words occurs before lexical selection takes place. The latter formulation fits the model of lexical representation in the bilingual brain suggested by de Bot [15] and de Groot [47]. According to this model, a common semantic system is connected to two independent lexical systems corresponding to each of the two languages of the bilingual individual.

**Figure 3:**
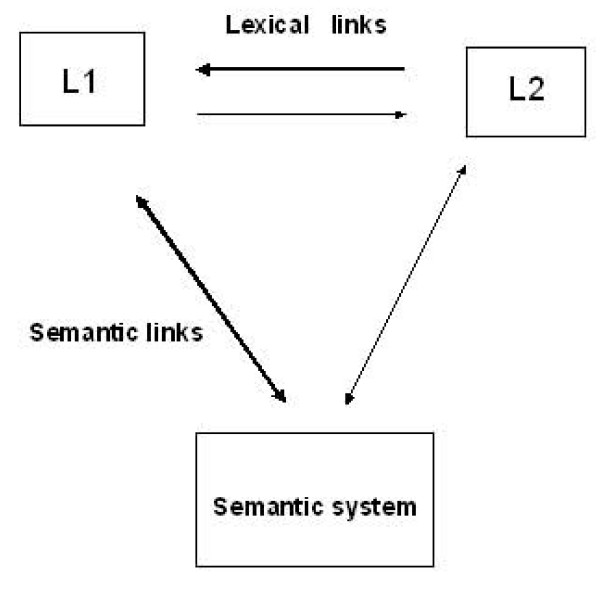
**Hierarchical model of bilingual lexical representation**. *Adapted from Kroll & Stewart *[18].

The ease of access to each lexicon from the semantic memory depends on factors such as fluency [48]. In other models the words of each language are interconnected and interact at the lexical level, and the access of a word in the second language to its meaning is often mediated by its translation equivalent in the first language [18]. Kroll and Stewart argued that the connections between translation words at the lexical level are asymmetric, but the strength of these connections is determined by the bilingual individual's proficiency in the second language [18] (see Figure 3).

**Figure 4:**
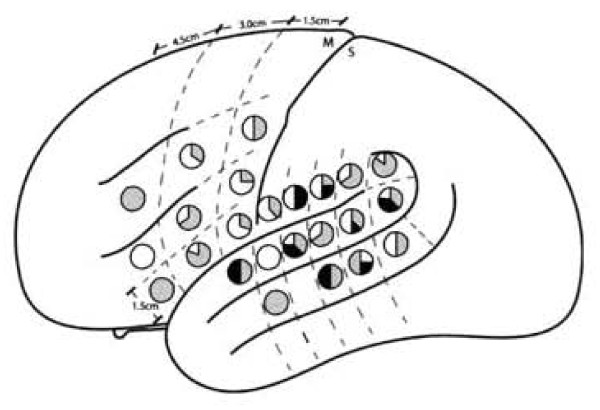
**Schematic figure depicting the proportion of significant sites by cortical zones**. Significant L1 sites are shown in gray, L2 sites in black, and shared sites in white. *Adapted from Lucas et al*. [11].

From a neurolinguistic viewpoint L1 and L2 modules, although similar in overall cortical extent, differ in anatomical distribution. Thus, for example, Lucas et al. found some brain sites which, upon stimulation, interfered with picture naming in both languages [11]. At the same time they also observed some sites which, when stimulated, disturbed naming in L1 but not in L2, and still other sites at which the opposite occurred. It thus seemed that posterior language regions might contain areas specific for L2 processing (see Figure 4).

Simos et al. studied receptive language areas in 11 bilingual patients with the aid of magnetic source imaging and found that in both languages there were differences in temporal lobe representations [2]. Temporal lobe receptive language centers for L1 and L2 have also been found to differ among people with late L2 acquisition. Recent research in speech perception suggests that there are differences in the phonetic perception of first and second languages (for reviews see [32,49]). It should be noted, however, that adaptation of the phonetic features of L2 is a necessary component of L2 speech acquisition; consequently, bilingual individuals who attain a high level of L2 proficiency are able to exploit the phonetic categories of that language in speech production and perception [50]. Further evidence for an assimilation process comes from a case study described by my group [33] in which a Russian-Hebrew bilingual aphasic woman showed a dissociation between her ability to perceive her second language (learned in adulthood) when it was spoken by a native speaker and when it was spoken by a speaker with an accent like her own. We interpreted this finding as support for the hypothesis that the assimilation procedure can be differentially damaged, such that L2 speech that conforms to L1 phonology (accented speech) is better perceived than phonemically correct L2 speech. This interpretation is in line with the interesting finding of dissociation between the speech production and the phonological abilities of our patient.

## Conclusion

Aphasia is a language disorder that results from damage to portions of the brain responsible for language. For most people, these are parts of the left hemisphere. However, data from the present investigation of MH, a bilingual aphasic patient, strongly support the existence of distinct language-specific cortical centers for certain first and second languages (Arabic-Hebrew), and are in line with a previous finding that posterior regions, including temporal and parietal lobes, possess language-specific sites for L2 [11]. Furthermore, this study is compatible with the hierarchical model of bilingual lexical representation suggested by Kroll & Stewart [18]. The perception deficits exhibited by MH suggest that bilingual individuals might possess two separate switching mechanisms: a lexical and a semantic mechanism. The findings of this case study provide evidence that Hebrew, as a second language, is represented in the brain by a subsystem that does not represent Arabic (the first language), and that this subsystem is more fragile, and therefore more sensitive to brain damage.

A limitation of this study is that the patient that took part in this study does not have equal level in language abilities (e.g. writing ability) as reflected by their different years of education. Due to previous findings of the differential effect of writing complexity on weak students' reading level versus students with good skills [51], generalization of the results to include students with dyslexia can not be reliable. Nevertheless, this case report might shed light on the relationship between language and certain neurobiological mechanisms, while also providing new evidence contributing to a better understanding of the dynamics of processing two languages in the bilingual brain.

## Abbreviations

BNT: Boston Naming Test; CT: computed tomography; fMRI: functional magnetic resonance imaging; ER-fMRI: event-related fMRI; L1: first language; L2: second language; WAB: Western Aphasia Battery; WCST: Wisconsin Card Sorting Cards test; SD: standard deviation.

## Competing interests

The author declares that they have no competing interests.

## Consent section

Written informed consent was obtained from the patient for publication of this case report and accompanying images.
